# Detection and Genotypic Characterization of Clindamycin Resistance Among *Staphylococcus aureus* Clinical Isolates

**DOI:** 10.1155/sci5/8532158

**Published:** 2026-08-31

**Authors:** Raechal Sanjana Hans, Suchitra Shenoy M.

**Affiliations:** ^1^ Department of Microbiology, Kasturba Medical College Mangalore, Manipal Academy of Higher Education, Manipal, India, manipal.edu

**Keywords:** clindamycin, *erm*, inducible resistance, *msrA*, *Staphylococcus aureus*

## Abstract

**Purpose:**

Resistance to macrolide, lincosamide, and streptogramin B (MLS_B_) antibiotics in *Staphylococcus aureus* has emerged as a significant therapeutic challenge. *erm* gene–encoded ribosomal methylation is expressed either constitutively or inducibly, causing resistance to erythromycin and clindamycin with possible induction of clindamycin resistance during therapy. *msrA*‐mediated active drug efflux confers resistance to macrolides and streptogramin B while preserving clindamycin susceptibility. This study aimed to determine the antimicrobial susceptibility pattern of *S. aureus* isolates and correlate between the phenotypic and genotypic patterns of clindamycin resistance.

**Methods:**

This cross‐sectional study was conducted from October 2022 to September 2023 at a tertiary care hospital laboratory. A total of 233 clinical isolates of *S. aureus* were included from exudate, blood, and bodily fluids. Antimicrobial susceptibility testing was performed using the Vitek 2 Compact system and interpreted according to CLSI 2022 and 2023 guidelines. Inducible clindamycin resistance was detected using the D‐test. Multiplex and uniplex PCR assays were performed for the detection of *ermA*, *ermB*, *ermC*, and *msrA* genes.

**Results:**

Among 233 *S. aureus* isolates, methicillin resistance was identified in 45.9% (107/233) isolates. The prevalence of constitutive MLS_B_ (cMLS_B_), inducible MLS_B_ (iMLS_B_), and MS phenotypes was 23.17%, 12.01%, and 10.3%, respectively. Overall clindamycin resistance was observed in 38.6% (90/233; 95% CI: 32.6%–45.0%). The *ermC* gene was detected in 30.47% (71/233) isolates, while *msrA* was identified in 9.44% (22/233), including 91.7% of isolates of the MS phenotype. Neither *ermA* nor *ermB* genes were detected. All isolates were susceptible to vancomycin, daptomycin, and tigecycline.

**Conclusion:**

*ermC*‐mediated MLS_B_ resistance was present in 30.5% of isolates, with cMLS_B_ (23.2%) being more common than iMLS_B_ (12.0%). Routine D‐testing is essential to avoid clinical failure when using clindamycin for erythromycin‐resistant, clindamycin‐susceptible *S. aureus*. Also, 8 isolates were clindamycin resistant and susceptible to erythromycin, showcasing the diversity of the resistance profile of *S. aureus* toward MLS_B._

## 1. Introduction

*Staphylococcus aureus* is a major human pathogen responsible for a wide spectrum of community‐ and hospital‐acquired infections, ranging from superficial skin and soft tissue infections to severe invasive diseases. Over the years, *S. aureus* has developed resistance to multiple classes of antibiotic classes, posing a significant therapeutic challenge worldwide [1].

Clindamycin is widely used as an empiric therapeutic agent for community‐acquired staphylococcal skin, soft tissue, bone, and joint infections because of its excellent bioavailability, tissue penetration, and its ability to suppress virulence factors like Panton–Valentine leukocidin (PVL), toxic shock syndrome toxin‐1 (TSST‐1), and alpha‐hemolysin [2, 3]. However, resistance to clindamycin among *S. aureus* isolates has increased considerably in recent years, thereby limiting its clinical utility [1, 2].

Resistance to macrolide–lincosamide–streptogramin B (MLS_B_) antibiotics in *S. aureus* is primarily mediated by methylation of the 23S ribosomal RNA target site encoded by *erm* genes [1]. Depending on gene expression, MLS_B_ resistance may occur as constitutive MLS_B_ (cMLS_B_) resistance or inducible MLS_B_ (iMLS_B_) resistance. In constitutive resistance, methylase enzymes are continuously produced, resulting in resistance to both erythromycin and clindamycin during routine susceptibility testing. In contrast, isolates with inducible resistance appear resistant to erythromycin but susceptible to clindamycin in vitro; however, exposure to macrolides during therapy may induce methylase production, leading to the emergence of clindamycin resistance and possible treatment failure [1, 2]. The predominant *erm* gene in *S. aureus* is *ermC* followed by *ermA* while *ermB* is near negligible [3]. Another important mechanism of resistance is active drug efflux mediated by the *msrA* gene among which *msrA* is only detected in *S. aureus*, which confers resistance to macrolide and streptogramin B antibiotics while retaining susceptibility to clindamycin, thereby producing the MS phenotype [4].

The increasing prevalence of methicillin‐resistant *S. aureus* (MRSA) together with MLS_B_ resistance further narrows the available treatment options for staphylococcal infections [2, 4, 5]. The present study aimed to determine the antimicrobial susceptibility profile of *S. aureus* isolates, with emphasis on correlation between phenotypic (D‐test) and genotypic resistance patterns associated with MLS_B_ antibiotics.

## 2. Materials and Methods

This cross‐sectional study was conducted in the Department of Microbiology, KMC, Mangalore, after the approval of the Institutional Ethics Committee, over 18 months from September 2022 to December 2023. The sample size of 227 was calculated assuming 18% of resistance to clindamycin among the *S. aureus* clinical isolates with 5% absolute precision and 95% confidence. All nonduplicate consecutive Gram‐positive cocci identified by the Vitek 2 Compact system as *S. aureus* isolated from blood, exudate, and body fluids between October 2022 and December 2022 were collected and preserved at −20°C in 20% glycerol broth. Of 233 *S. aureus* isolates, 132 (56.65%) were recovered from pus, 62 (26.60%) from blood, 12 (5.15%) from deep tissue, 24 (10.3%) from wound swabs, and 1 (0.42%) each from ascitic fluid, bone, and nasal swab specimens. The antibiotic susceptibility pattern was extracted from microbiological records. *S. aureus* isolates were analyzed for detection of resistance toward MLS_B_ antibiotics by phenotypic and genotypic methods [6, 7].

### 2.1. Antibiotic Susceptibility Testing (AST)

AST of *S. aureus* isolates was phenotypically tested using MIC determination in the Vitek 2 Compact system using the Vitek 2 AST‐P628 card and interpreted as per CLSI guidelines M100, 2022 and 2023 [8, 9]. The quality control strains of *S. aureus* ATCC 29213, ATCC BAA‐976, and ATCC BAA‐977 were used to ensure the quality check of the Vitek testing. All *S. aureus* isolates resistant to erythromycin were evaluated for clindamycin resistance by the D‐test with both erythromycin (15 μg) and clindamycin (2 μg) disks placed 15 mm apart and incubated aerobically at 37°C for 24 h [6]. Results were interpreted as 3 different phenotypes of *S. aureus.*

### 2.2. Molecular Methods

DNA extraction: DNA extraction of purified colonies of *S. aureus* was performed using the boiling method, and the extracts were kept frozen at −20°C until use [6, 7]. The primer sequence and the conditions (Table 1) were followed as per those described by Nikbakht et al. [6].

**TABLE 1:** PCR thermocycler conditions.

Target gene	Primer sequence	Amplicon size (bp)	PCR type	Thermocycler conditions
*ermA*	F: TATCTTATCGTTGGAAGGGATT R: CTACACTTGGCTTAGGATGAAA	139	Multiplex	• Initial denaturation at 94°C for 5 min • 30 cycles of denaturation at 95°C for 1 min, annealing at 55°C for 30 s, and extension at 72°C for 1 min • Final extension at 72°C for 5 min
*ermB*	F: CTATCTGATTGTTGAAGAAGGATT R: TTTACTCTTGGTTTAGGATGAAA	142		
*ermC*	F: CTTGTTGATCACGATAATTTCC R: ATCTTTTAGCAAACCCGTATTC	190		

*msrA*	F: TCCAATCATTGCACAAAATC R: AATTCCCTCTATTTGGTGGT	163	Uniplex	• Initial denaturation at 94°C for 5 min. • 30 cycles of denaturation at 95°C for 1 min, annealing at 52°C for 30 s, and extension at 72°C for 1 min. • Final extension at 72°C for 5 min

If the isolates were positive by multiplex PCR, uniplex PCR was performed for each gene individually to confirm the gene as the base pair sizes of all three genes range from 139 bp to 190 bp and may overlap during gel documentation.

For the PCR assay targeting macrolide resistance (*erm*) genes, *S. aureus* ATCC BAA‐977 was used as the positive control for *ermA,* while *S. aureus* ATCC BAA‐976, was used as the positive control for *ermC* detection. For uniplex PCR targeting the *msrA* gene, *S. aureus* ATCC BAA‐976 carrying the *msrA* gene was used as the positive control. *S. aureus* ATCC 25923 served as the negative control [6], and a reagent control containing all PCR components without template DNA was included in all PCR runs.

### 2.3. Statistical Analysis

Statistical analyses were performed using Jamovi software Version 2.4.14. Descriptive statistics were used to summarize the distribution of *S. aureus* isolates, antimicrobial susceptibility patterns, inducible and constitutive clindamycin resistance phenotypes, and resistance genotypes. Associations between categorical variables were evaluated using the chi‐square test or Fisher’s exact test, as appropriate. Odds ratios (ORs) with 95% confidence intervals (CIs) were calculated to estimate the strength of associations between phenotypic and genotypic characteristics. To account for multiple comparisons, the Bonferroni correction was applied, and the threshold for statistical significance was adjusted accordingly (*α* = 0.05/*n*, where *n* represents the number of comparisons performed). A two‐sided *p* value less than the Bonferroni‐adjusted significance level was considered statistically significant.

## 3. Results

**FIGURE 1:**
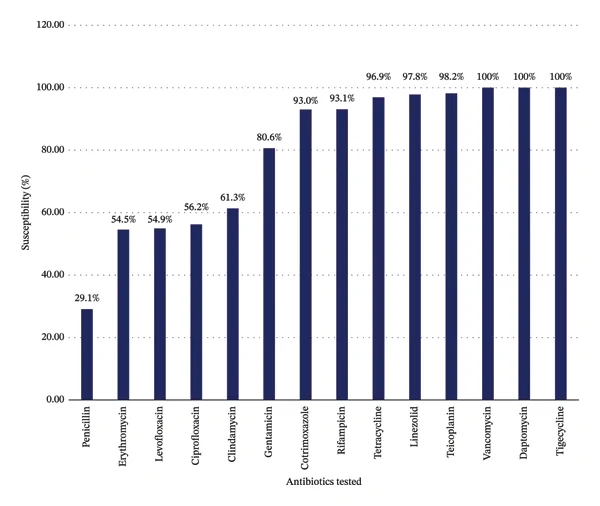
Antibiotic susceptibility pattern of *Staphylococcus aureus* isolates (*n* = 233).

A total of 233 *S. aureus* isolates were included in the study. All isolates were susceptible to vancomycin, daptomycin, and tigecycline followed by teicoplanin (98.2%), linezolid (97.8%), tetracycline (96.9%), cotrimoxazole (93%), rifampicin (93.1%), and gentamicin (80.6%). The susceptibility to penicillin (29.1%), ciprofloxacin (56.2%), and levofloxacin (54.9%) was lowest (Figure 1). A total of 107 isolates were MRSA (45.49%).

While cMLS_B_ was seen in 54 isolates and iMLS_B_ in 28 isolates, 8 isolates were clindamycin resistant with erythromycin sensitive; the total number of isolates resistant to clindamycin was 90 (38.6%). Erythromycin resistance was seen in 106 isolates (45.49%).

**FIGURE 2:**
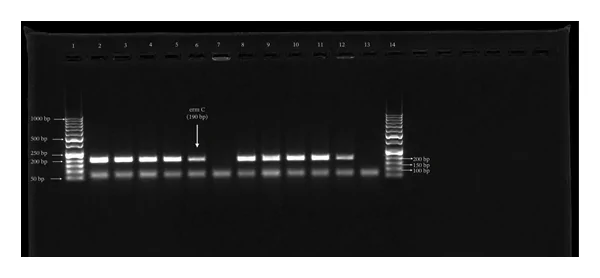
Detection of *erm* genes among *S. aureus* isolates by multiplex PCR. Lane 1: 50‐bp DNA ladder, Lane 2: positive control for *ermC*—*S. aureus* ATCC BAA‐976, Lanes 3–6: test isolates with *ermC* gene (190 bp), Lane 7: isolate without *erm* genes, Lanes 8–12: test isolates with *ermC* gene (190 bp), Lane 13: negative control for *ermA, ermB,* and *ermC* genes—*S. aureus* ATCC 25923, Lane 14: 50‐bp DNA ladder.

**FIGURE 3:**
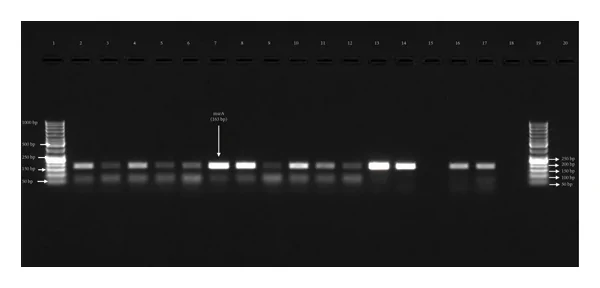
Detection of *msrA* gene among *S. aureus* isolates by uniplex PCR. Lane 1: 50‐bp DNA ladder, Lane 2: positive control *S. aureus* ATCC BAA‐976 having *msrA* gene, Lanes 3–14: test isolates with *msrA* gene (163 bp), Lane 15: test isolate without *msrA* gene, Lanes 16–17: test isolates with *msrA* gene (163 bp), Lane 18: negative control for *msrA* gene—*S. aureus* ATCC 25923, Lane 19: 50‐bp DNA ladder, Lane 20: reagent control.

The *ermC* gene (Figure 2) was detected in 71/233 (30.47%) isolates, of which 22 were iMLS_B_ and 49 were cMLS_B_ isolates. *msrA* gene (Figure 3) prevalence was 22/233 (9.44%) overall and 22/24 (91.7%) among MS phenotype isolates. *ermA* and *ermB* genes were not detected in any of the 233 isolates included in the study (0/233).

## 4. Discussion

*S. aureus* infections are usually treated with the beta‐lactam group of drugs, and clindamycin may be added to the list to reduce the impact of the toxin produced, especially in skin and soft tissue infections. In the case of MRSA strains, caution must be taken to avoid beta‐lactam drugs, and drugs such as teicoplanin, vancomycin, and linezolid are used. In our study, all the strains were susceptible to vancomycin, daptomycin, and tigecycline. Susceptibility to teicoplanin (98.2%), linezolid (97.8%), tetracycline (96.9%), cotrimoxazole (93%), rifampicin (93.1%), and gentamicin (80.6%) was maintained. Decreased susceptibility to penicillin (29.1%), ciprofloxacin (56.2%), and levofloxacin (54.9%) was observed. MRSA constituted 45.9% (107/233) of isolates, similar to reports from Africa [10]. The nearly equal distribution of MRSA and MSSA suggests stabilization of methicillin resistance, possibly due to heterogeneous antibiotic selection pressure and ongoing clonal turnover in community‐healthcare interface settings [11–13]. Vancomycin susceptibility was comparable to findings from Nepal [14], although a study from eastern India [15] reported 11.6% resistance.

In the present study, the erythromycin resistance rate was 45.49% (106/233; OR 0.83; 95% CI: 39.1%–51.9%), which is higher than rates reported from several Indian studies, where erythromycin resistance and associated MLS_B_ resistance patterns ranged between 25% and 30% in regions such as Maharashtra, Karnataka, Andhra Pradesh, and Tamil Nadu [10, 11, 16]. Comparatively lower resistance rates of 15%–22% were reported globally from studies conducted in the United States, Brazil, and Israel, indicating substantial geographic variation in macrolide resistance patterns [2, 17–19].

The overall clindamycin resistance rate observed in the present study was 38.6% (90/233; 95% CI: 32.6%–45.0%) compared with rates of approximately 25%–30% reported in previous studies from South India (25%, *p* < 0.001; 30%, *p* = 0.004) and 15%–22% from studies conducted in the United States, Brazil, and Israel (all *p* < 0.001) [18, 20–22]. This difference is probably due to ongoing selective pressure [10] and region‐specific antimicrobial prescribing practices.

In the current investigation, constitutive clindamycin resistance was found in 54/233 (23.17%; OR 1.30; 95% CI: 18.2%–29.0%) *S. aureus* isolates. In India, cMLS_B_ rates ranged from 20% to 27% in Delhi, Maharashtra, Karnataka, Tamil Nadu, and Andhra Pradesh [11–13]. This contrasts with the higher occurrence in regions such as Greece, northwestern Iran, and Haryana, India (60%, 37.3%, and 34.42%) in the studies reported in the years 2010–2022 [6, 13]. Constitutive clindamycin resistance was more frequent among MRSA isolates (59.25%) than MSSA isolates (40.74%) (*p* < 0.001; Bonferroni‐adjusted *p* < 0.003, OR 2.11; 95% CI 1.25–3.57). Similar findings have been reported previously [1, 6, 18, 20, 23], although MLS_B_ resistance patterns vary with geographic region, strain type, methicillin susceptibility, infection source, age, and antimicrobial usage. The high prevalence of cMLS_B_ among MRSA highlights the need for cautious clindamycin use and consideration of alternative agents guided by susceptibility testing.

The frequency of inducible clindamycin resistance in the present study was 28/233 (12.01%; OR 0.14; 95% CI: 8.1%–16.9%; *p* < 0.001) and was consistent with earlier studies from other regions of India and worldwide [11–13]. Our study findings showed a higher frequency of inducible clindamycin resistance among MSSA isolates (57.14%) than MRSA isolates (42.85%); however, the association was not statistically significant (*p* = 0.22; Bonferroni‐adjusted *p* = 0.66; [OR]1.15; 95% CI: 0.52–2.55). Contrast findings were observed worldwide between 2005 and 2022 [13, 16, 20, 23], highlighting the importance of region‐specific surveillance for guiding empirical clindamycin therapy. The absence of a significant association indicates that methicillin susceptibility does not exclude inducible clindamycin resistance, supporting routine D‐test screening for both MSSA and MRSA isolates. However, nonperformance of the D‐test screening may guide inappropriate clindamycin selection, particularly in MRSA isolates, and result in therapeutic failure and complications of the infection.

The MS phenotype was identified in 10.3% (24/233; OR 0.11; 95% CI: 6.7%–14.9%) of *S. aureus* isolates, comparable to reports from Maharashtra (10.5%, *p* = 0.92) and South India (10.5%; *p* = 0.92) in 2020 [11, 16], while the global prevalence has been reported 5.7%–44.8% [6, 11, 16]. Such differences in MS_B_ phenotype rates, associated with the *msrA* gene, emphasize the importance of performing the D‐test in distinguishing true clindamycin susceptibility from inducible MLS_B_ resistance to ensure appropriate therapy. The MS phenotype was more frequent among MRSA isolates (79.16%) than MSSA isolates (20.8%) in the present study (*p* < 0.001; Bonferroni‐adjusted *p* < 0.003, OR 5.23; 95% CI 1.88–14.55), consistent with findings from Brazil [18] and Africa [1]. The predominance of the MS phenotype among MRSA isolates is clinically beneficial, as it preserves clindamycin as an effective, orally bioavailable, and cost‐effective outpatient therapeutic option.

A broader One Health perspective may further contextualize the observed resistance patterns. *S. aureus*, the predominant pathogen causing bovine subclinical mastitis (88.4% of culture‐positive milk samples), has been shown to exhibit resistance to multiple antimicrobial agents. Furthermore, hand milking has been identified as a risk factor for mastitis, highlighting the contribution of human–animal interactions to staphylococcal transmission. These findings suggest that livestock may serve as reservoirs of antimicrobial‐resistant *S. aureus*, facilitating dissemination across human and veterinary interfaces and emphasizing the importance of integrated One Health surveillance strategies [24].

Genotypic analysis demonstrated the presence of *ermC* and *msrA* genes in 30.47% (71/233; OR 0.44; 95% CI: 24.8%–36.7%; *p* < 0.001) and 9.44% (22/233; OR 0.10; 95% CI: 6.1%–13.9%; *p* < 0.001) of isolates, respectively. Previous studies from India and globally identify *ermC* as the predominant mediator of MLS_B_ resistance, likely reflecting propagation of regional *ermC*‐harboring clones, while *msrA* remains infrequently reported [5, 11, 16, 17, 19, 21, 25]. The absence of *ermA* and *ermB* genes (0/233; 95% exact CI: 0%–1.6%) in the present study is consistent with contemporary evidence indicating that *ermC,* a plasmid‐coded resistance determinant, is the predominant mediator of MLS_B_ resistance in *S. aureus*. *ermA*, a chromosomally encoded gene, is generally reported at lower frequencies than *ermC. ermB,* which is typically carried on plasmids or transposable genetic elements, is infrequently detected in *S. aureus* and more commonly associated with streptococci and enterococci and has not become widely established among circulating *S. aureus* clones. Although horizontal gene transfer can facilitate the acquisition of *ermB*, its limited dissemination within successful *S. aureus* lineages may explain the absence of both *ermA* and *ermB* in the present study [3]. These findings emphasize the importance of region‐specific molecular surveillance and indicate that the observed resistance patterns are largely clone‐driven, reinforcing the need for locally tailored antimicrobial stewardship and clindamycin‐use policies.

A subset of cMLS_B_ and iMLS_B_ isolates did not harbor the *ermA*, *ermB*, or *ermC* genes investigated (Table 2). Since MLS_B_ resistance is predominantly mediated by ribosomal methylases encoded by *erm* genes, these findings may indicate the presence of other untested *erm* determinants, sequence variations affecting primer binding, or alternative resistance mechanisms. Further studies involving expanded gene screening and sequencing are warranted to identify the underlying genetic basis of resistance. Similarly, in two MS phenotypes, the *msrA* gene was not detected, with the possibility of other macrolide‐resistant genes like *msrB* and *mphC*. Two isolates susceptible to erythromycin and clindamycin exhibited the *msrA* gene. This genotype–phenotype discordance may highlight that detection of gene may not result in phenotype expression or the expression is too low to note the resistance above the breakpoints.

**TABLE 2:** Correlation between the phenotype and genotype presentation of clindamycin resistance.

	*ErmC*	*MsrA*	No genes	Total
cMLS_B_	49	—	5	54
iMLS_B_	22	—	6	28
MS	—	22	2	24
Clindamycin sensitive	—	2	117	119
Only clindamycin resistant	—	—	8	8
Total	71	24	138	233

## 5. Limitation

The study did not screen for *lnu(A)* and *lnu(B)*, which confer lincosamide‐specific resistance without macrolide resistance. This may account for the 8 isolates (3.4%) with a clindamycin‐resistant, erythromycin‐sensitive phenotype in the present study. *lnu* genes have been briefly discussed in other studies and are most likely responsible for this resistance, underscoring the variable and diverse nature of clindamycin resistance in *S. aureus* [22].

## 6. Conclusion

In our study, *ermC*‐mediated MLS_B_ resistance was present in 30.5% of isolates, with cMLS_B_ (23.2%) being more common than iMLS_B_ (12.0%). The D‐test remains an indispensable phenotypic assay for the accurate detection of MLS_B_ resistance phenotypes in *S. aureus*, thereby enabling judicious clindamycin utilization and minimizing the likelihood of therapeutic failure. Routine incorporation of the D‐test into antimicrobial susceptibility testing protocols is imperative for optimized antimicrobial stewardship and improved clinical outcomes.

## Author Contributions

Raechal Sanjana Hans: data curation, resources, formal analysis, and writing–original draft.

Suchitra Shenoy M.: conceptualization, data curation, formal analysis, methodology, project administration, resources, supervision, writing–original draft, and review and editing.

## Funding

This research received no specific grant from any funding agency in the public, commercial, or not‐for‐profit sectors.

## Disclosure

The study was presented as a poster at the 51st Annual National Conference of the Indian Association of Preventive and Social Medicine (IAPSMCON 2024) held from February 8 to 10, 2024, at Kasturba Medical College in Mangaluru.

## Ethics Statement

Ethics approval for this study was obtained from the Institutional Ethics Committee of Kasturba Medical College, Mangalore, Ref. No: IEC KMC MLR IEC/2023/045.

## Conflicts of Interest

The authors declare no conflicts of interest.

## Data Availability

The data that support the findings of this study are available from the corresponding author upon reasonable request.
